# Efficacy and safety of tripterygium glycosides combined with ACEI/ARB on diabetic nephropathy: a meta-analysis

**DOI:** 10.3389/fphar.2024.1493590

**Published:** 2025-01-17

**Authors:** Zhuan’E. Yao, Pengbo Wang, Qinjuan Fu, Qiong Song, Haojian Xu, Peng Zhang

**Affiliations:** ^1^ Department of Nephrology, Shaanxi Provincial People’s Hospital, Xi’an, Shaanxi, China; ^2^ Department of Nephrology, Shaanxi Provincial Second People’s Hospital, Xi’an, Shaanxi, China

**Keywords:** diabetic nephropathy, tripterygium glycosides, ACEI/ARB, meta-analysis, efficacy

## Abstract

**Aims:**

This study aims to evaluate the efficacy and safety of tripterygium glycosides combined with angiotensin-converting enzyme inhibitors/angiotensin receptor blockers (ACEI/ARBs) in treating Diabetic nephropathy and provide high-level evidence to support its standardized application.

**Methods:**

Literatures were retrieved from PubMed, Web of Science, EMBASE, Cochrane Library, CNKI, Wanfang and VIP databases, the search time frame was defined as from the time of establishment to April 2023. This study only included randomized controlled trials of tripterygium glycosides combined with ACEI/ARB in the treatment of diabetic nephropathy, and the final included studies were identified according to the inclusion and exclusion criteria, and meta-analysis of data was performed using RevMan 5.3 software.

**Results:**

A total of 44 RCTs with 3537 DN patients were included in the study. Compared with the control group, tripterygium glycosides combined with ACEI/ARB significantly reducing 24 h-UTP (24 h urine total protein) [SMD = −1.46, 95% CI (−1.70, −1.23), P < 0.00001], increasing effective rate [RR = 1.23, 95% CI (1.17,1.29), P < 0.00001], elevating serum albumin [SMD = 0.85, 95% CI (0.69, 1.02), P < 0.00001], improving serum creatinine [SMD = −0.35, 95% CI (−0.59, −0.11), P = 0.004], with no difference in BUN (blood urea nitrogen) [SMD = −0.17, 95% CI (−0.48,0.13), P = 0.27], the adverse reactions rate was higher than those of the control group [RR = 1.96, 95%CI (1.43, 2.68), P < 0.0001].

**Conclusion:**

This study showed that the combination of tripterygium glycosides and ACEI/ARB was more effective than ACEI/ARB alone. However, the side effects of the combined treatment group were higher than those of the control group, especially liver function damage, which also suggested that its safety in the treatment of diabetic nephropathy was worth considering. Therefore, although tripterygium glycosides provided a choice for the clinical treatment of diabetic nephropathy, its side effects limited its clinical application. In future studies, we need to further optimize tripterygium glycosides and reduce its side effects to ensure the safety of clinical application.

## 1 Introduction

Diabetic nephropathy (DN) is a common microvascular complication of diabetes and the common causes of chronic kidney disease (CKD), which is mainly characterized by proteinuria and decreased glomerular filtration rate. It seriously affects the quality of life of patients and brings huge economic burden to patients and society, so more attention should be paid to DN (Liu et al., 2023; Chen et al., 2020). There is currently no radical treatment for diabetic nephropathy, so it is important to prevent or alleviate the disease at the early stage. Clinically, glycemic control, blood pressure control, reducing cholesterol levels, the adjustment of unhealthy lifestyle and the use of angiotensin-converting enzyme inhibitor (ACEI) or angiotensin II receptor antagonist (ARB) are often used to slow disease progression (Pillai and Fulmali, 2023; Chen et al., 2022). In recent years, studies have also shown that sodium-glucose co-transporter 2 inhibitors (SGLT2i) can significantly improve the renal outcomes and reduce the risk of renal failure and cardiovascular events in patients with type 2 diabetes, glucagon-like peptide-1 receptor agonists (GLP-1 RAs) has a better renal protection effect in DN patients and fineronone is effective and safe in the treatment of diabetic nephropathy (Perkovic et al., 2019; Shaman et al., 2022; Bakris et al., 2015). Although these measures have shown certain efficacy in the treatment of DN, many patients still progress to end-stage renal disease (ESKD) and even have to undergo renal replacement therapy such as hemodialysis or peritoneal dialysis. It is necessary to find more new effective therapies to prevent the disease.

In recent years, traditional Chinese medicine (TCM)has shown its unique advantages in the treatment of diabetic nephropathy, many studies have shown that the application of TCM in patients with diabetic nephropathy can delay its progression to end-stage renal disease and reduce the mortality rate of patients (Chen et al., 2019; Liu et al., 2022). As an immunosuppressant, tripterygium wilfordii is a traditional Chinese medicine, originally written in the “Shennong’s Herbal Classic”, belongs to the Celastraceae family, which is widely used in the treatment of rheumatic diseases and glomerulonephritis in China. The main active metabolites of tripterygium wilfordii are tripterygium wilfordii polyglycosides, terpenoids, glycosides, etc (Liu et al., 2021). Tripterygium glycosides can stabilize the permeability of the glomerular basement membrane, reduce proteinuria, protect podocyte and reduce podocyte damage in patients with DN (Lengnan et al., 2020). In recent years, tripterygium glycosides combined with angiotensin-converting enzyme inhibitors/angiotensin receptor blockers (ACEI/ARBs) is also reported to be effective in treating diabetic nephropathy in China, however, the effectiveness of these studies is not consistent, and the reports on safety are not comprehensive. Therefore, this study conducted a meta-analysis on the basis of existing RCT studies, aiming to lay a foundation for evaluating whether TG combined with ACEI/ARB plays a more prominent role in the treatment of diabetic nephropathy and provide evidence-based medical evidence for clinical application.

## 2 Materials and methods

### 2.1 Search strategy

Literature was retrieved from PubMed, Web of Science, EMBASE, Cochrane Library, CNKI, Wanfang and VIP databases, the search time frame was defined as from the time of establishment to April 2023. The following keywords were used in the literature search:“diabetic nephropathy”, “diabetic kidney diseases”, “diabetic glomerulosclerosis”, “tripterygium glycosides”, “tripterygium wilfordii”, “tripterygium wilfordii Hook F”.

### 2.2 Inclusion and exclusion criteria

Inclusion criteria are as follows: (1) All subjects were diagnosed as diabetic nephropathy; (2) All included studies were randomized controlled trials studies; (3) The experimental group was patients treated with tripterygium glycosides in combination with ACEI/ARB, the control group consisted of patients treated with ACEI/ARB alone.

Exclusion criteria are as follows: (1) Incomplete original data; (2) Lack of rigorous experimental design; (3) Repeated studies, case reports, animal or cell experiments; (4) Non-randomized controlled study; (5) Missing data or primary results.

### 2.3 Outcome indicators

The main outcome indicators include 24-hour urine total protein (24 h-UTP). The secondary outcome indicators include total effective rate (number of effective cases/total number of cases), serum albumin (ALB), blood creatinine (Scr), blood urea nitrogen (BUN) and adverse events.

### 2.4 Research data extraction

The extracted data included the first author, year of publication, sample size, intervention, duration of treatment, outcome indicators and adverse events. Studies were screened by 2 independent reviewers based on inclusion and exclusion criteria to exclude ineligible studies, and in case of disagreement between reviewers, the disagreement was resolved through discussion or consensus reached on the basis of screening results obtained by the third reviewer.

### 2.5 Assessment on risk of bias

Quality assessment of the included studies according to the assessment criteria in the Cochrane Handbook of Systematic Evaluation, including the following 7 items: (1) Random sequence generation; (2) allocation concealment; (3) blinding of participants and personnel; (4) blinding of outcome assessment; (5) incomplete outcome data; (6) selective reporting; (7) other biases.

### 2.6 Statistical analysis

We applied Review Manager 5.3 software and Stata version 17.0 (Stata Corp, College Station, TX, USA) to statistically analyze the data from the included studies. Risk Ratios (RR) was used for dichotomous variables, standardized mean difference (SMD) for continuous variables, and 95% confidence interval (CI) was used for all variables. We applied the Cochran-Q test and I^2^ statistic to assess the heterogeneity of included studies. If I^2^ was below 50%, the data were analyzed using a fixed-effects model. If I^2^ was above 50%, the data were analyzed using a random effects model. Publication bias was analyzed by funnel plot and evaluated quantitatively by egger’s test.

## 3 Results

### 3.1 Literature search results

**FIGURE 1:**
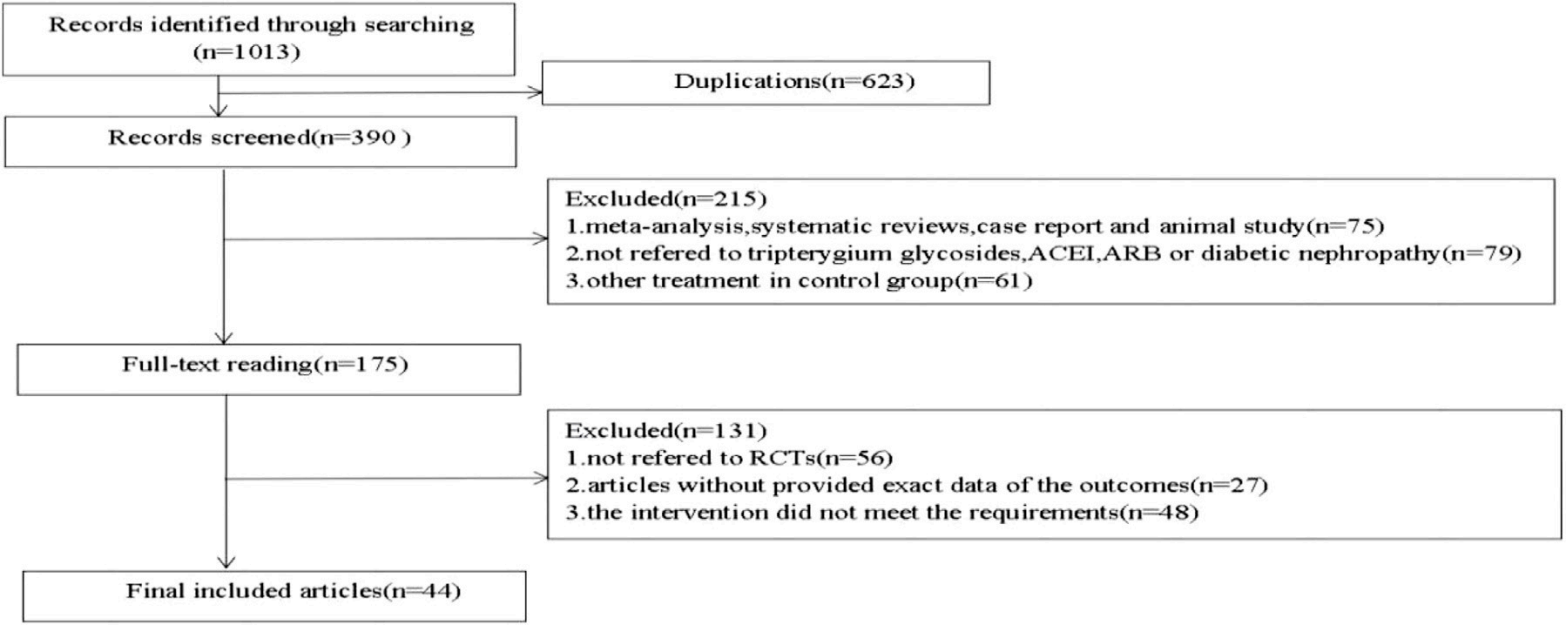
Research selection process.

Initially, 1,013 relevant articles were identified out by seven databases. By reading titles and abstracts, duplicate literature, animal experiments, reviews, cell studies and case reports were excluded. In the remaining literature, by reading the full text, we excluded the literature that did not meet the requirements of the experimental design, missing data, and no interventions. Finally, a total of 44 literature were included in this study. The research selection process is shown in Figure 1.

### 3.2 Quality of included studies

**FIGURE 2:**
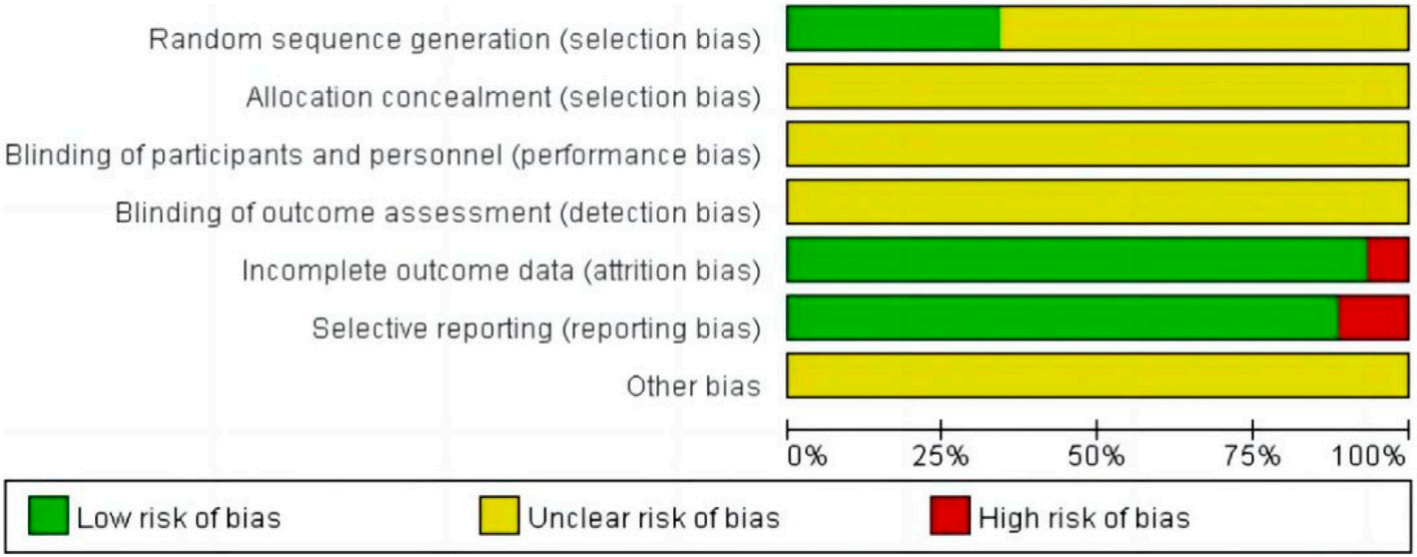
Risk of bias graph: review authors’ judgements about each risk of bias item presented as percentages across all included studies.

We applied the Cochrane Collaborations tool to assess the quality of the included studies. 44 studies [12–55] included were all RCTs, of which 15 articles mentioned the method of randomization (random number table method was used in 11 articles, computer randomization method was used in 2 articles, random envelope method was used in 1 article, and random drawing method was used in 1 article). 29 studies only mentioned randomization without specifying the method of randomization, all the articles did not mentioned allocation concealment, and no study mentioned other biases. The results of the quality assessment are shown in Figure 2.

### 3.3 Trial characteristics

The 44 RCT studies included a total of 3,537 patients,1785 in the experimental group, and 1752 in the control group. The baseline data were balanced between the two groups of patients, and the basic characteristics are shown in Table 1.

**TABLE 1:** Characteristics of the studies included in the meta-analysis.

Study	N (E/C)	Male (E/C)	Age (E/C)	DN staging (E/C)	Disease duration (E/C)	Treatment		Outcomes	Duration (week)
						Experimental group	Control group		
Cai (2012)	32/29	20/18	50.4 ± 4.7/49.9 ± 3.5	V	9.1 ± 1.78/8.9 ± 1.8	TG 20 mg tid + Valsartan 40–80 mg/d	Valsartan 40–80 mg/d	24 h-UTP, Effective rate, SCr, ALB, Adverse reaction	24
Cao (2010)	30/30	—	—	—	—	TG 40 mg qd + Valsartan 80 mg bid	Valsartan 160 mg qd	24 h-UTP, SCr, BUN, Adverse reaction	12
Chang et al. (2020)	62/62	43/45	50.3 ± 11.8/49.6 ± 12.3	—	—	TG 60 mg/d + Valsartan 160 mg/d	Valsartan 160 mg/d	24 h-UTP, SCr, ALB, Adverse reaction	24
Chen et al. (2012)	25/25	12/11	57.3 ± 11.6/56.9 ± 12.1	—	13.3 ± 3.0/12.0 ± 4.0	TG 40 mg qd + Irbesartan 150 mg qd	Irbesartan 150 mg qd	24 h-UTP, SCr, BUN, ALBAdverse reaction	12
Chen, (2013)	20/20	13/20	59.2 ± 9.83/55.1 ± 11.7	IV-V	10.6 ± 2.3/10.1 ± 3.9	TG 1.0 mg/kg bid or tid + Benazepril 10 mg bid	Benazepril 10 mg bid	24 h-UTP, Effective rate	4
Chi (2013)	32/32	20/18	46.3 ± 10.2/45.1 ± 11.3	III-IV	—	TG 20–30 mg tid + Benazepril	Benazepril	24 h-UTP, SCr, BUN, ALB, Adverse reaction	4
Diao et al. (2013)	25/25	14/13	48–65/49–63	III	0.2–7/0.3–6	TG 20–30 mg tid + Fosinopril 10 mg/d-20 mg/d	Fosinopril 10 mg/d-20 mg/d	24 h-UTP, SCr, ALB, Adverse reaction	12
He et al. (2010)	31/29	15/16	58.2 ± 10.83/59.8 ± 11.43	—	9.13 ± 5.77/9.65 ± 4.58	1–8 weeks:TG 20 mg tid 9–24 weeks:TG 10 mg tid + Valsartan 80–160 mg/d	Valsartan 80–160 mg/d	24 h-UTP, SCr, ALB, Adverse reaction	24
He (2016)	35/35	24/25	57.8 ± 6.9/58.5 ± 6.8	≥IV	9.2 ± 1.3/9.5 ± 1.2	TG 1.0 mg/kg bid or tid + Benazepril 5 mg bid	Benazepril 5 mg bid	24 h-UTP, Effective rate, Adverse reaction	8
Jiang (2018)	46/46	27/26	58.4 ± 11.2/57.3 ± 10.1	—	11.4 ± 7.1/10.5 ± 6.5	TG 0.3–0.5 mg/kg tid + Valsartan 80 mg qd	Valsartan 80 mg qd	24 h-UTP, Effective rate, SCr	3
Jiang (2015)	62/64	32/36	51.7 ± 7.6/50.9 ± 8.7	II-IV	6.75 ± 1.68/6.91 ± 1.32	TG 20 mg tid + Telmisartan 80 mg qd	Telmisartan 80 mg qd	24 h-UTP, Effective rate, SCr, BUN	12
Jia et al. (2020)	26/26	15/16	60.79 ± 3.31/60.82 ± 3.37	—	—	TG 0.3–0.5 mg/kg tid + Valsartan 80 mg bid	Valsartan 80 mg bid	24 h-UTP, SCr, ALB, Adverse reaction	12
Jin and Mei (2020)	50/50	24/23	61.75 ± 4.63/61.37 ± 4.68	IV	6.58 ± 1.06/6.22 ± 1.14	TG 1.0 mg/kg·d tid + Valsartan 80 mg qd	Valsartan 80 mg qd	24 h-UTP, SCr, ALB, Adverse reaction	8
Li (2014)	48/48	31/30	52.7 ± 2.8/51.9 ± 2.5	—	4.12 ± 0.35/4.06 ± 0.38	1–12 weeks:TG 20 mg tid 13–24 weeks:TG 10 mg tid + Irbesartan 75 mg qd-150 mg bid	Irbesartan 75 mg qd-150 mg bid	24 h-UTP, Effective rate, SCr, ALB	24
Li (2011)	20/20	11/10	52.0 ± 4.0/60.0 ± 7.2	IV	9.0 ± 3.4/8.0 ± 2.6	TG 20 mg tid + Valsartan	Valsartan	24 h-UTP, Effective rate, BUN, SCr, ALB, Adverse reaction	14
Lin and Li (2018)	31/31	20/17	37.66 ± 5.98/38.41 ± 6.25	—	6.0–13.0/8.0–11.0	1–12 weeks:TG 20 mg tid 13–24 weeks:TG 10 mg tid + Candesartan 8 mg qd	Candesartan 8 mg qd	24 h-UTP, SCr, BUN, ALB, Adverse reaction	24
Liu (2015)	42/42	23/22	51.17 ± 6.91/51.83 ± 6.47	—	3.06 ± 1.52/3.12 ± 1.38	1–8weeks:TG 20 mg tid 9–24weeks:TG 20 mg qd + Valsartan 160 mg qd	Valsartan160 mg qd	24 h-UTP, SCr	24
Li et al. (2015)	27/26	16/17	55.37 ± 9.97/56.12 ± 10.34	—	—	TG 20 mg tid + Irbesartan 150 mg/d	Irbesartan 150 mg/d	24 h-UTP, Effective rate, SCr, ALB	24
Lu (2017)	25/25	14/15	56.3 ± 5.1/57.2 ± 5.8	—	—	TG 20 mg tid + Valsartan 160 mg qd	Valsartan 160 mg qd	24 h-UTP, SCr, ALB	12
Pu et al. (2013)	98/98	45/48	51.12 ± 12.43/53.65 ± 11.09	—	8.25 ± 2.17/8.78 ± 2.56	TG 20 mg tid + Valsartan 80 mg qd	Valsartan 80–160 mg qd	24 h-UTP, Effective rate	4
Shen (2014)	90/90	51/46	59.8 ± 13.1/59.1 ± 12.8	I-IV	14.7 ± 9.4/13.7 ± 9.9	TG 20 mg tid + Irbesartan 150 mg bid	Irbesartan 150 mg bid	24 h-UTP, Effective rate	2
Song et al. (2005)	35/32	21/22	50.1 ± 10.5/53.1 ± 11.3	—	8.9 ± 5.3/10.6 ± 5.8	TG 1.0–2.0 mg/kg·d + Benazepril 5–20 mg	Benazepril 5–20 mg	24 h-UTP, SCr, ALB	24
Song (2014)	40/40	22/24	51.24 ± 5.77/50.33 ± 5.32	V	7.45 ± 3.28/7.45 ± 3.28	TG 20 mg tid + Valsartan 40 mg qd	Valsartan 40 mg qd	24 h-UTP, Effective rate, SCr, BUN, Adverse reaction	24
Tan and Wang (2010)	25/23	16/15	52/49.8	—	3.9/4.2	1–12 weeks:TG 20 mg tid 13–24 weeks:TG 10 mg tid + Irbesartan 75 mg qd-150 mg bid	Irbesartan 75 mg qd-150 mg bid	24 h-UTP, Effective rate, ALB, Adverse reaction	24
Tu et al. (2017)	108/108	66/62	51.2 ± 5.9/52.3 ± 6.3	III-IV	6.1 ± 2.3/6.3 ± 2.1	TG 1.5 mg/kg·d tid + Telmisartan 40 mg qd	Telmisartan 40 mg qd	24 h-UTP, Effective rate, SCr, BUN	4
Wang and Zhu (2019)	55/55	33/35	59.13 ± 3.64/58.79 ± 3.42	—	7.09 ± 2.35/7.35 ± 2.16	TG 1.5 mg/kg·d tid + Benazepril 5 mg bid	Benazepril 5 mg bid	24 h-UTP, Effective rate	12
Wang (2017a)	30/30	13/12	49.1 ± 4.6/48.4 ± 4.2	—	—	TG 0.5 mg/kg tid + Benazepril 10 mg qd	Benazepril 10 mg qd	24 h-UTP, Effective rate	2
Wang (2016)	26/26	14/15	50.4 ± 13.4/51.0 ± 13.7	—	0.5–10/0.5–12	TG 0.3–0.5 mg/kg tid + Valsartan 80 mg qd	Valsartan 80 mg qd	24 h-UTP, Effective rate	12
Wang and Zhang (2012)	52/30	38/19	39–67/41–65	—	2.5–11.7/2.8–10.6	1–8 weeks:TG 20 mg tid 9–24 weeks:TG 20 mg qd + Valsartan 160 mg qd	Valsartan 160 mg qd	24 h-UTP, SCr, ALB, Adverse reaction	24
Wang (2017b)	45/45	30/29	58.01 ± 6.85/57.89 ± 7.01	IV-V	9.2 ± 1.29/9.17 ± 1.32	TG 1.0 mg/kg bid or tid + Benazepril 5 mg bid	Benazepril 5 mg bid	24 h-UTP, Effective rate, Adverse reaction	8
Wang and Xiao (2013)	32/30	19/16	38–67/35–65	—	4.5–18/5–18	1–8 weeks:TG 20 mg tid 9–24 weeks:TG 10 mg tid + Telmisartan 40–80 mg/d	Telmisartan 40–80 mg/d	24 h-UTP, SCr, BUN	24
Wu (2010)	32/30	20/19	48.0 ± 25.6/49.6 ± 26.2	—	—	TG 40 mg tid + Telmisartan 80 mg bid	Telmisartan 80 mg bid	24 h-UTP, SCr, ALB, Adverse reaction	12
Wu et al. (2012)	33/32	—	47.89 ± 5.92/46.75 ± 5.64	—	5–19/5–20	1–8weeks:TG 20 mg tid 9–24weeks:TG 10 mg tid + Valsartan 80–160 mg qd	Valsartan 80–160 mg qd	24 h-UTP, Effective rate, SCr, ALB, Adverse reaction	24
Wu and Shi (2018)	34/34	19/18	55 ± 9/55 ± 8	IV	9.3 ± 1.8/8.7 ± 1.9	1–8weeks:TG 20 mg tid 9–24weeks:TG 10 mg tid + Valsartan 80–160 mg/d	Valsartan 80–160 mg/d	24 h-UTP, SCr, BUN, ALB, Adverse reaction	24
Xu (2014)	25/25	16/17	47.5 ± 3.1/46.9 ± 3.2	—	7.4 ± 1.2/7.1 ± 1.3	TG 1.0 mg/kg tid + Benazepril 10 mg bid	Benazepril 10 mg bid	24 h-UTP, Effective rate, SCr, BUN	4
Xu et al. (2016)	20/20	12/11	61.21 ± 2.51/59.85 ± 3.38	IV	—	TG 1.0 mg/kg·d tid + Valsartan 80 mg qd	Valsartan 80 mg qd	24 h-UTP, SCr, ALB, Adverse reaction	8
Yang (2016)	14/16	8/8	—	IV	—	TG 1.0 mg/kg tid + Losartan 50 mg qd	Losartan 50 mg qd	24 h-UTP, SCr	8
Yang et al. (2018)	25/25	16/15	59.24 ± 6.28/59.65 ± 6.33	—	7.14 ± 3.33/7.08 ± 3.23	TG 1.0 mg/kg tid + Benazepril 5 mg bid	Benazepril 5 mg bid	24 h-UTP, Effective rate	4
Yan (2017)	46/46	26/25	42.0 ± 4.3/41.9 ± 4.3	—	6.3 ± 2.6/6.3 ± 2.6	TG 20 mg tid + Valsartan 80–160 mg qd	Valsartan 80–160 mg qd	24 h-UTP, Effective rate, SCr, Adverse reaction	12
Zhang (2015)	66/65	46/40	59.94 ± 6.53/59.82 ± 6.79	IV	13.30 ± 1.95/13.24 ± 2.03	TG 30 mg bid + Valsartan 80 mg qd	Valsartan 80 mg qd	24 h-UTP, Effective rate, SCr, Adverse reaction	48
Zhang et al. (2012)	50/50	35/33	53.66 ± 7.80/54.23 ± 8.23	—	13.23 ± 6.52/14.35 ± 7.12	1–12 weeks:TG 20 mg tid 13–24 weeks:TG 10 mg tid + Irbesartan 75 mg qd-150 mg bid	Irbesartan 75 mg qd-150 mg bid	24 h-UTP, Effective rate, SCr, ALB	24
Zhao et al. (2011)	23/23	14/13	58.6 ± 5.4/59.4 ± 6.2	—	1.25–6.5/1.1–6.4	TG 20 mg tid + Valsartan 160 mg qd	Valsartan 160 mg qd	24 h-UTP, SCr, ALBAdverse reaction	12
Zhou, (2013)	15/15	10/11	35–68/39–70	—	—	TG 40 mg tid + Valsartan 80–160 mg/d	Valsartan 80–160 mg/d	24 h-UTP, SCr, ALB, Adverse reaction	8
Zhou et al. (2017)	97/99	62/68	63.5 ± 8.4/61.7 ± 10.2	—	13.0 ± 4.1/13.2 ± 3.3	TG 20 mg tid + Valsartan 160 mg qd	Valsartan 160 mg qd	24 h-UTP, SCr, BUN, ALB, Adverse reaction	4

### 3.4 Meta-analysis results

#### 3.4.1 24 h urine total protein (24 h-UTP)

All the included studies [12–55] reported changes of 24 h-UTP after treatment between the experimental group and control group. The random-effect model was adopted to analyze the data due to the heterogeneity (P < 0.00001, I^2^ = 89%), and the results showed that the experimental group was more effective in reducing 24 h-UTP than the control group [SMD = −1.46, 95% CI (−1.70–1.23), P < 0.00001].

**FIGURE 3:**
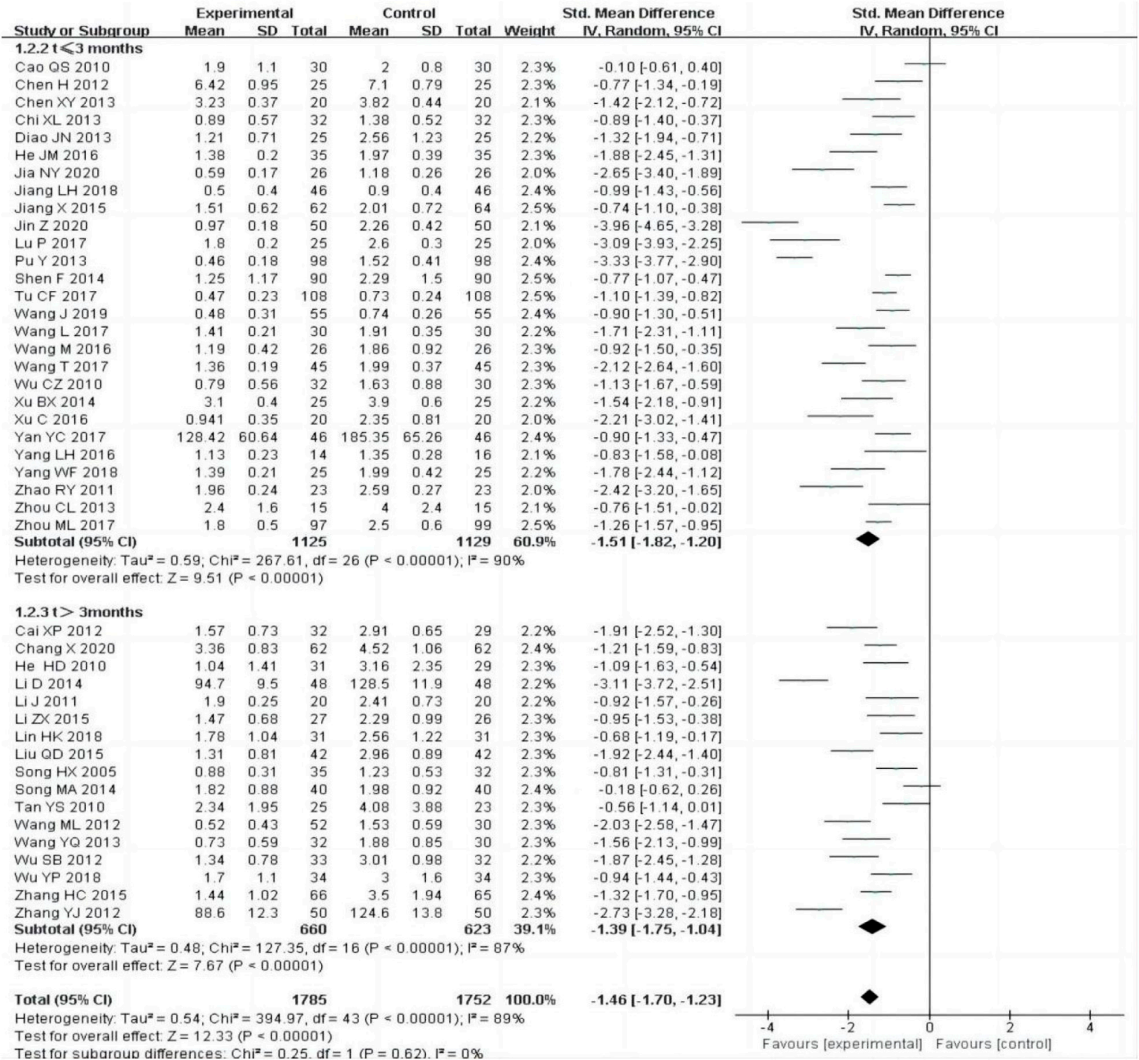
Forest plots of 24-h UTP after treatment.

The subgroup was divided into t ≤ 3 months and t > 3 months, the heterogeneity was still obvious within each subgroup [t ≤ 3 months: P < 0.00001, I^2^ = 90%; t > 3 months:P < 0.00001, I^2^ = 89%], subgroup analysis also showed that the experimental group was superior to the control group in reducing 24 h-UTP [Figure 3].

**FIGURE 4:**
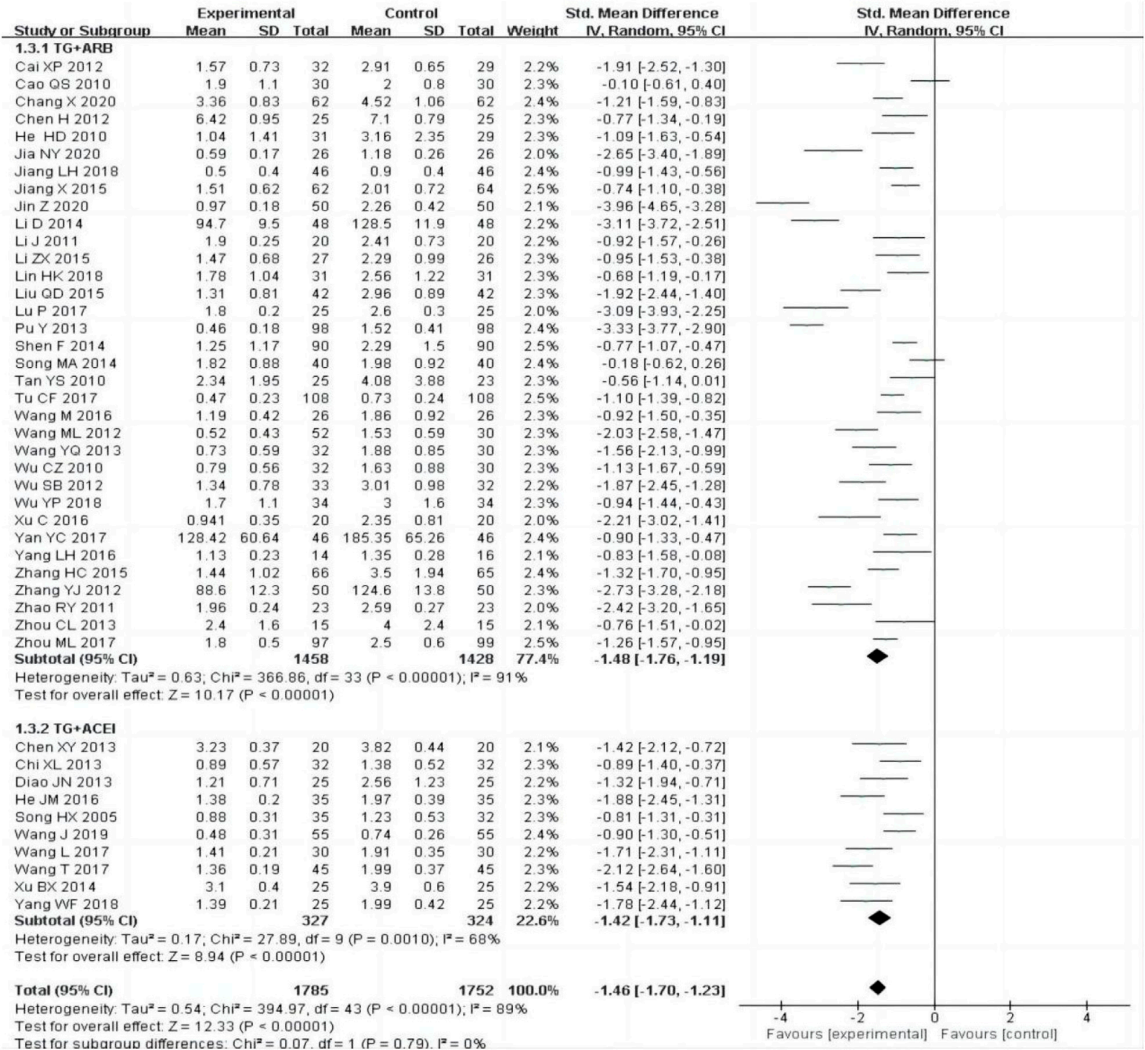
Forest plots of 24-h UTP after treatment.

The subgroup was divided into TG + ARB and TG + ACEI, the heterogeneity was still obvious within each subgroup [TG + ARB:P < 0.00001, I^2^ = 91%; TG + ACEI:P = 0.001, I^2^ = 68%], subgroup analysis also showed that the experimental group was superior to the control group in reducing 24 h-UTP [Figure 4].

#### 3.4.2 Effective rate

**FIGURE 5:**
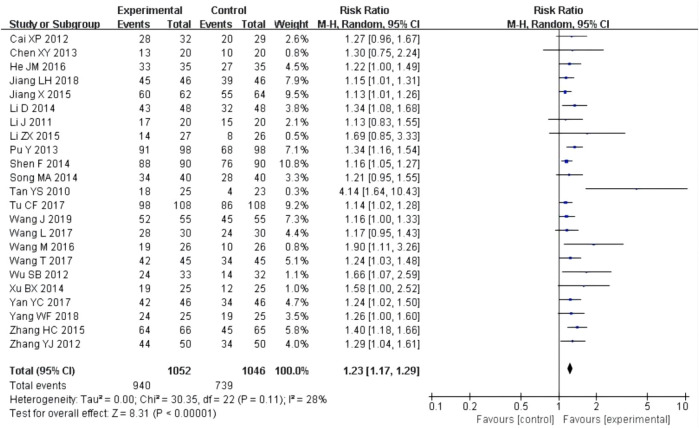
Forest plots of effective rate after treatment.

23 studies (Cai, 2012; Chen, 2013; He, 2016; Jiang, 2018; Jiang, 2015; Li, 2014; Li, 2011; Li et al., 2015; Pu et al., 2013; Shen, 2014; Song, 2014; Tan and Wang, 2010; Tu et al., 2017; Wang and Zhu, 2019; Wang L., 2017; Wang, 2016; Wang T., 2017; Wu et al., 2012; Xu, 2014; Yang et al., 2018; Yan, 2017; Zhang, 2015; Zhang et al., 2012) reported effective rate after the treatment. Although the heterogeneity test (P = 0.11, I^2^ = 28%) showed no significant heterogeneity, the confidence interval with the fixed-effect model was very narrow, so the random-effect model was used. The results showed that the experimental group had a better effect rate than the control group [RR = 1.23, 95% CI (1.17, 1.29), P < 0.00001, Figure 5].

#### 3.4.3 Serum albumin (ALB)

**FIGURE 6:**
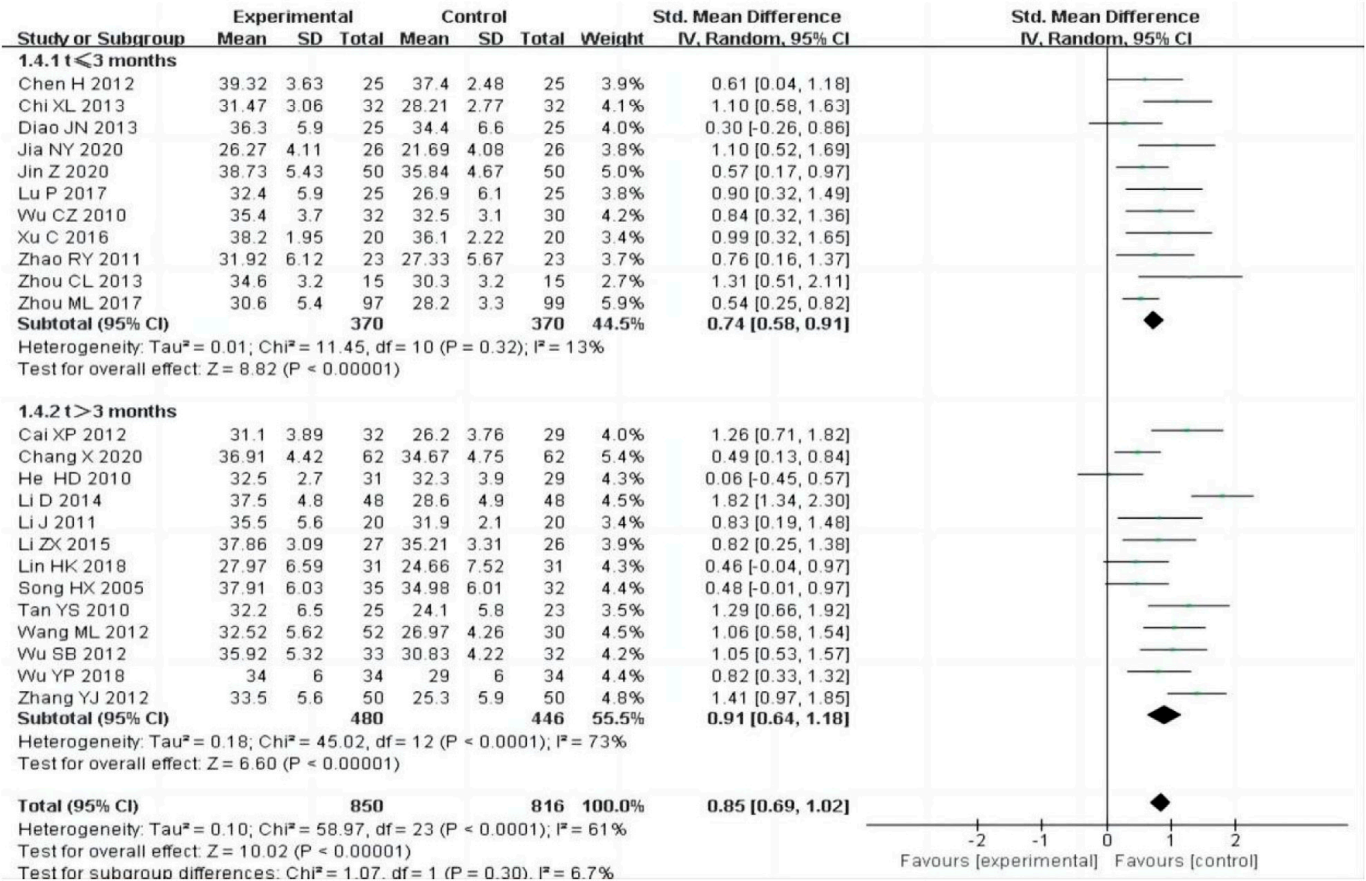
Forest plots of ALB after treatment.

24 studies (Cai, 2012; Chang et al., 2020; Chen et al., 2012; Chi, 2013; Diao et al., 2013; He et al., 2010; Jia et al., 2020; Jin and Mei, 2020; Li, 2014; Li, 2011; Lin and Li, 2018; Li et al., 2015; Lu, 2017; Song et al., 2005; Tan and Wang, 2010; Wang and Zhang, 2012; Wu, 2010; Wu et al., 2012; Wu and Shi, 2018; Xu et al., 2016; Zhang et al., 2012; Zhao et al., 2011; Zhou, 2013; Zhou et al., 2017) including 1,666 participants, reported ALB outcome after treatment, The random-effect model was adopted to analyze the data due to the heterogeneity (P < 0.0001, I^2^ = 61%), and the results showed that the experimental group was more effective at increasing ALB than the control group [SMD = 0.85, 95% CI (0.69, 1.02), P < 0.00001, Figure 6].

We performed subgroup analysis based on the treatment time, the subgroup was divided into t ≤ 3 months and t > 3 months, the result showed that the experimental group had a better curative effect than the control group (Figure 6).

#### 3.4.4 Serum creatinine (Scr)

**FIGURE 7:**
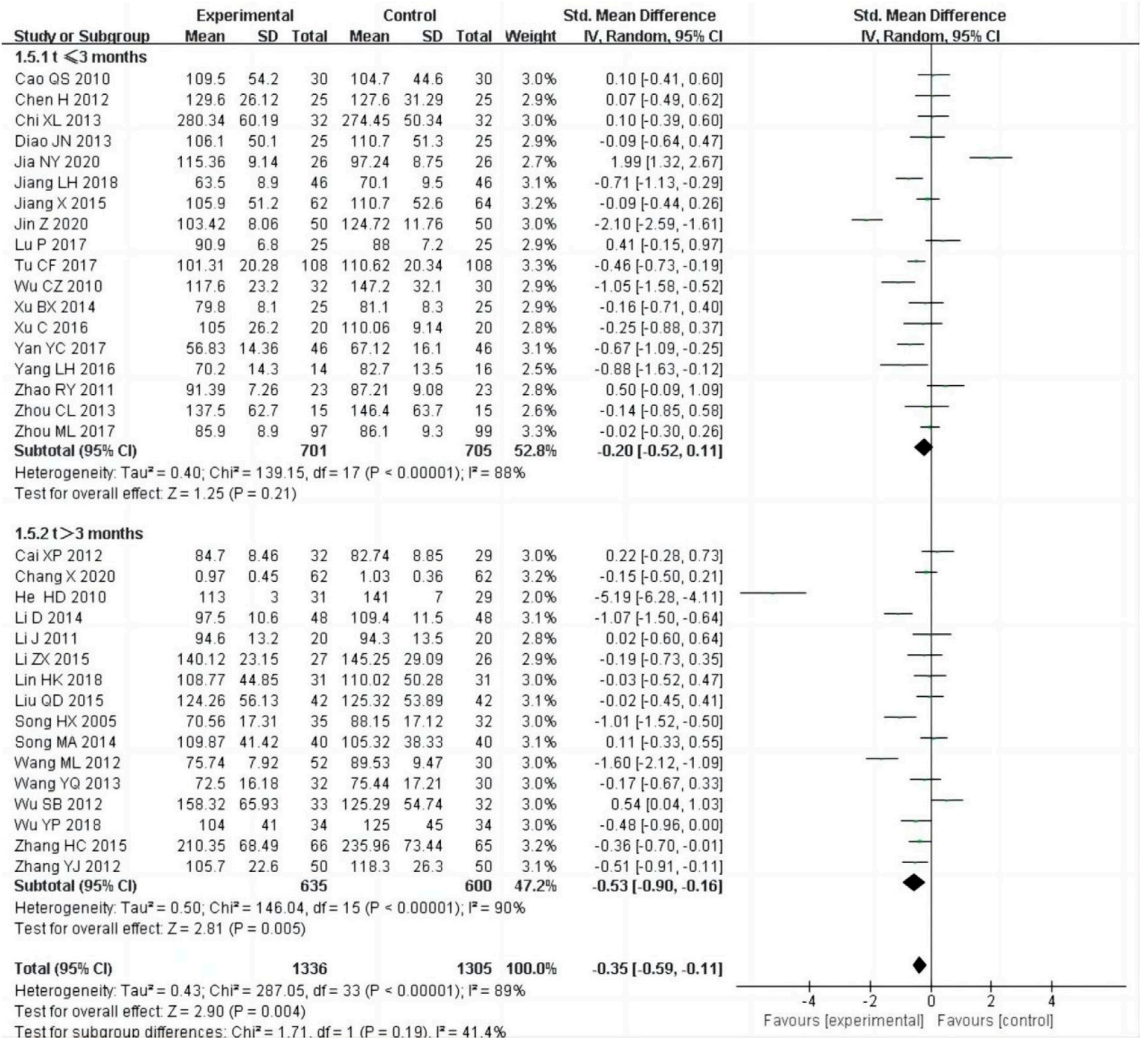
Forest plots of Scr after treatment.

34 studies (Cai, 2012; Cao, 2010; Chang et al., 2020; Chen et al., 2012; Chi, 2013; Diao et al., 2013; He et al., 2010; Jiang, 2018; Jiang, 2015; Jia et al., 2020; Jin and Mei, 2020; Li, 2014; Li, 2011; Lin and Li, 2018; Liu, 2015; Li et al., 2015; Lu, 2017; Song et al., 2005; Song, 2014; Tu et al., 2017; Wang and Zhang, 2012; Wang and Xiao, 2013; Wu, 2010; Wu et al., 2012; Wu and Shi, 2018; Xu, 2014; Xu et al., 2016; Yang, 2016; Yan, 2017; Zhang, 2015; Zhang et al., 2012; Zhao et al., 2011; Zhou, 2013; Zhou et al., 2017), including 2,641 patients, reported of Scr as an outcome. The random-effect model was adopted to analyze the data due to the heterogeneity (P < 0.0001, I^2^ = 89%), and the result showed that the experimental group could improve SCr better than control group [SMD = −0.35, 95% CI (−0.59, −0.11), P = 0.004, Figure 7]. We performed subgroup analysis based on the treatment time, the heterogeneity was still obvious within each subgroup [t ≤ 3 months:P < 0.00001, I^2^ = 88%; t > 3 months: P < 0.00001, I^2^ = 90%], subgroup analysis showed that there was no difference in SCr between the experimental group and control group, if the treatment duration was less than 3 months [SMD = −0.20, 95% CI (−0.52,0.11), P = 0.21, Figure 7], but if the treatment duration was longer than 3 months, there was a significant difference between the experimental group and control group [SMD = −0.53, 95% CI (−0.90, −0.16), P = 0.005, Figure 7].

#### 3.4.5 Blood urea nitrogen (BUN)

**FIGURE 8:**
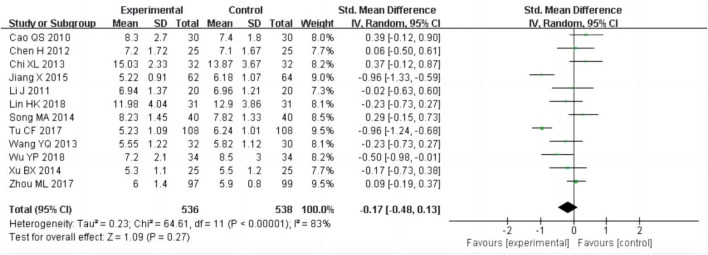
Forest plots of BUN after treatment.

12 studies (Cao, 2010; Chen et al., 2012; Chi, 2013; Jiang, 2015; Li, 2011; Lin and Li, 2018; Song, 2014; Tu et al., 2017; Wang and Xiao, 2013; Wu and Shi, 2018; Xu, 2014; Zhou et al., 2017), including 1,074 patients, reported of BUN as an outcome. As the heterogeneity test (P^2^ = 83%) showed significant heterogeneity, the random-effect model was used for this meta-analysis. The result indicated that there was no significant difference in reducing BUN between the experimental group and the control group [SMD = −0.17, 95% CI (−0.48, 0.13), P = 0.27, Figure 8].

#### 3.4.6 Adverse reactions

**FIGURE 9:**
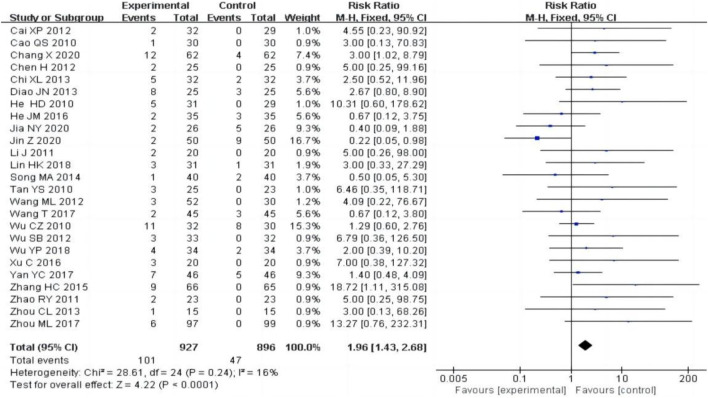
Forest plots of adverse reactions after treatment.

25 studies (Cai, 2012; Cao, 2010; Chang et al., 2020; Chen et al., 2012; Chi, 2013; Diao et al., 2013; He et al., 2010; He, 2016; Jia et al., 2020; Jin and Mei, 2020; Li, 2011; Lin and Li, 2018; Song, 2014; Tan and Wang, 2010; Wang and Zhang, 2012; Wang T., 2017; Wu, 2010; Wu et al., 2012; Wu and Shi, 2018; Xu et al., 2016; Yan, 2017; Zhang, 2015; Zhao et al., 2011; Zhou, 2013; Zhou et al., 2017), including 1823 patients, reported adverse reactions, the heterogeneity test (P = 0.24, I^2^ = 16%) showed no significant heterogeneity, the fixed-effect model was used for meta-analysis. The result showed that the experimental group had a higher adverse reactions rate than the control group [RR = 1.96, 95% CI (1.43, 2.68), P < 0.0001, Figure 9]. Of the 25 studies reporting adverse reactions, 101 cases in the experimental group had adverse reactions, including 45 cases (44.6%) of hepatic impairment, 11 cases (10.9%) of leukocytopenia, 13 cases (12.9%) of menstrual disorders and other adverse reactions. In the control group, there were 47 cases of adverse reactions, including 3 cases (6.4%) of hepatic impairment, 4 cases (8.5%) of leukocyte reduction, and no adverse reactions of menstrual disorders. In patients who developed liver function impairment, aminotransferase is mostly slightly elevated, which can be restored to the normal range with the addition of hepatoprotective drugs, such as polyene phosphatidylcholine, silymarin, compound glycyrrhizin and bifendate pills. For the adverse reactions of leukopenia and menstrual disorders, 8 original literatures only mentioned that the adverse reactions disappeared after symptomatic treatment, and 6 original literatures did not mention treatment measures. None of the original literatures reported in detail on the degree of severity and the specific measures taken.

#### 3.4.7 Sensitivity analysis and publication bias

**FIGURE 10:**
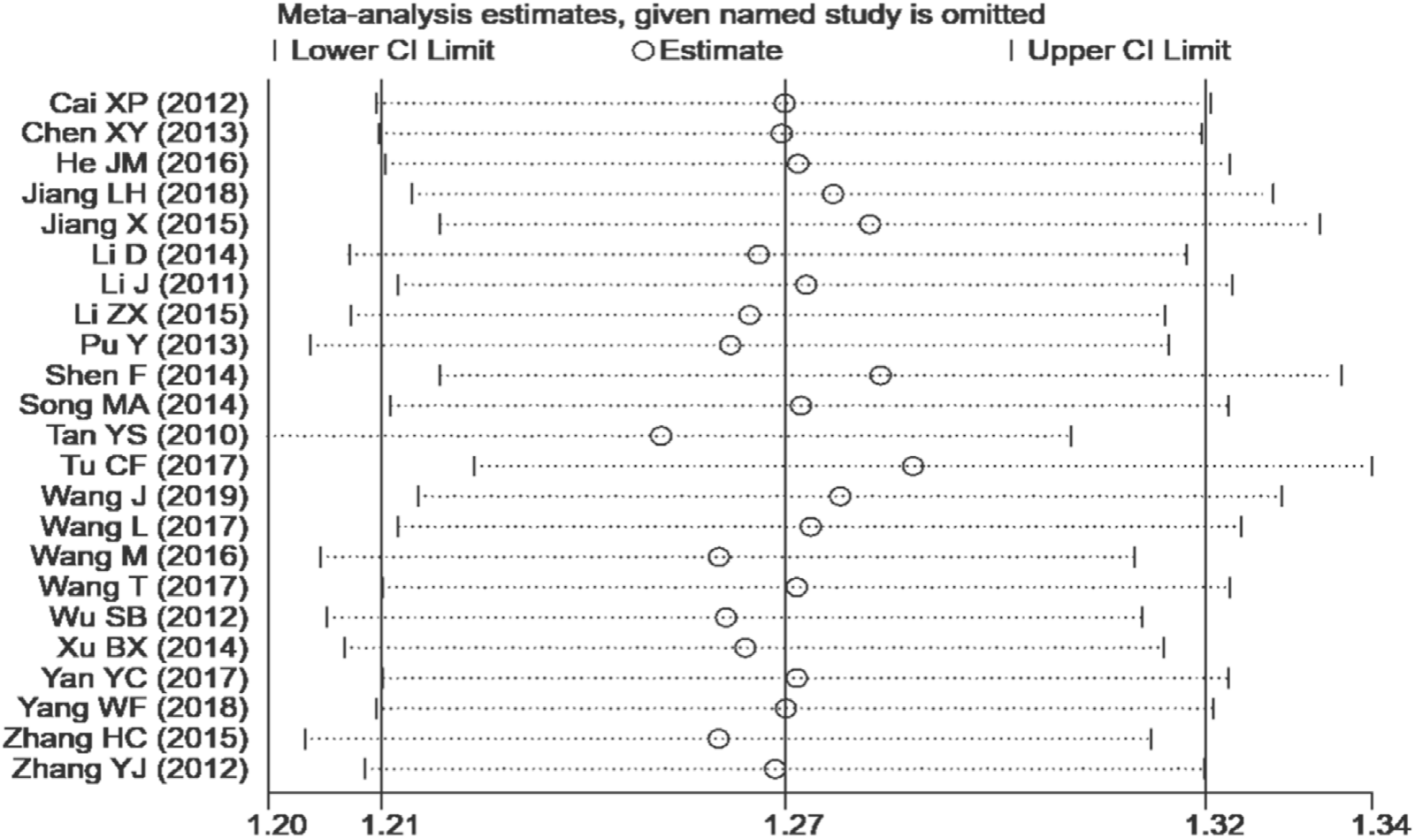
Sensitivity analysis on effective rate.

**FIGURE 11:**
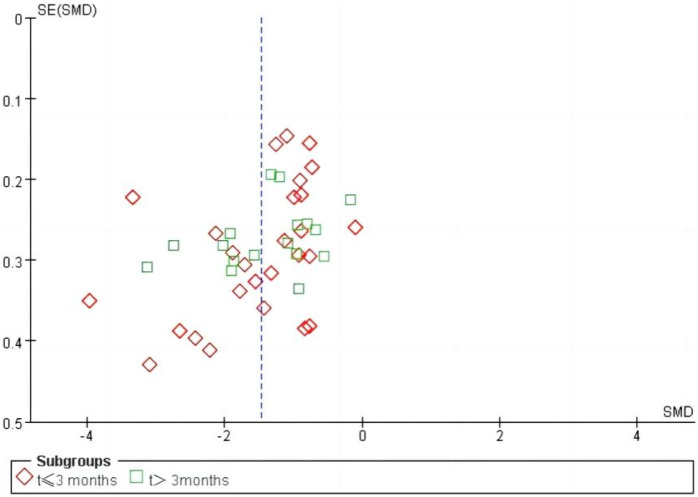
Funnel plot of publication bias of 24 h-UTP.

**FIGURE 12:**
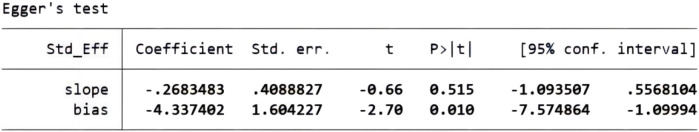
Egger’s test of 24 h-UTP.

We performed a sensitivity analysis on effective rate (Figure 10), and the results showed that no single article affected the overall analysis result, indicating that the results of this study were relatively stable. We used funnel plots to assess publication bias on 24 h-UTP, as shown in Figure 11, the funnel plot shows the presence of publication bias, the Egger’s test for 24 h-UTP (Figure 12) shows P = 0.010, which is consistent with funnel plot results, the publication bias is considered to be related to the heterogeneity of the study and the quality of the included articles.

## 4 Discussion

Diabetic nephropathy (DN) is the main cause of end-stage renal disease (ESRD). In recent years, the incidence of diabetic nephropathy has been gradually increasing due to the increasing incidence of diabetes patients. The pathogenesis of DN includes alterations in glomerular hemodynamics, increased activity of the renin-angiotensin system (RAS), genetic predisposition, oxidative stress, overexpression of inflammatory cytokines and ultimately development of glomerular sclerosis and renal failure (Giglio et al., 2023; Martini et al., 2008). As an important clinical feature of DN, persistent albuminuria is an independent risk factor of DN progression (Liu et al., 2021; Wu et al., 2017). Due to the complex pathogenesis of diabetic nephropathy, it is difficult to slow down the progression of the disease with the current conventional treatment methods. In recent years, studies have demonstrated that sodium-glucose cotransporter-2 (SGLT-2) inhibitors have a renal protective effect and reduce the incidence of cardiovascular events in patients with diabetic nephropathy (DKD) (Perkovic et al., 2019; Bhatt et al., 2021; Cherney et al., 2023). However, the potential adverse reactions of SGLT-2 inhibitors, such as increasing the risk of genital fungal infection and euglycaemic diabetic ketoacidosis in patients with type 2 diabetes, limit its clinical application (Liew et al., 2023). Some studies in China have shown that traditional Chinese medicine combined with Western medicine has better efficacy in the treatment of diabetic nephropathy. Tripterygium glycosides, as a traditional Chinese medicine, is also used in the treatment of diabetic nephropathy. Tripterygium glycosides can protect and reverse the damage of podocyte cytoskeleton, reduce podocyte damage, and decrease urinary protein (Zheng et al., 2008). A prospective, randomized, controlled clinical trial have shown that tripterygium glycosides can significantly reduce urine protein level in DN patients (Ge et al., 2013). A meta-analysis of 23 RCTs showed that low-dose tripterygium glycosides had a positive effect on the treatment of type 2 diabetic nephropathy, however, the inclusion criteria were broader, and the control group was given conventional treatment, or ACEI/ARB or metformin was given in addition to conventional treatment (Chen et al., 2022). The results of a study by Wang D et al. showed that tripterygium glycosides had good renoprotective effects, reducing urine protein, blood urea nitrogen and creatinine levels in patients with kidney disease, and had a good safety profile (Wang et al., 2018). Tripterygium glycosides is proved to not only regulate inflammatory factors such as TNF-α, IL-1β and TGF-β1, but also ameliorate podocyte injury and glomerular hypertrophy, reduce urinary protein in rats with diabetic nephropathy through anti-inflammation and inhibition of macrophage infiltration (Ma et al., 2013; Huang et al., 2020). A study showed that tripterygium glycosides may delay the transdifferentiation of renal tubulointerstitial cells to MyoF by down-regulating the expression of ACt-A, thus alleviating renal tubulointerstitial fibrosis, reducing urinary protein and protecting renal function (Zhao et al., 2014). It is well known that podocyte injury plays an important role in the pathogenesis of DN, which promotes the progression of diabetic nephropathy. Apoptosis and epithelium-mesenchymal transformation are the main causes of podocyte injury in diabetic nephropathy. Many studies have shown that over expression of twist1 increases apoptosis of podocytes, and tripterygium glycosides may reduce apoptosis and protect podocytes by inhibiting twist1 signaling pathway (Tao et al., 2021). It is worth noting that some studies have also reported that tripterygium glycosides has no definite efficacy on reducing serum creatinine, urea nitrogen and increasing blood albumin in diabetic nephropathy (Chen et al., 2012; Wang and Xiao, 2013). Therefore, in this meta-analysis, 44 studies were included to explore the efficacy and safety of tripterygium glycosides combined with ARB/ACEI in diabetic nephropathy. In order to better evaluate the efficacy and safety of tripterygium glycosides in the treatment of diabetic nephropathy, we selected the effective rate, 24 h-UTP, ALB, Scr, BUN and adverse reactions for statistical analysis. By analyzing several outcome indexes, we found that compared with ACEI/ARB treatment alone, tripterygium glycosides combined with ACEI/ARB could improve the effective rate, increase plasma albumin and improve renal function in patients with diabetic nephropathy, suggesting that the effect of TG combined with ACEI/ARB in treating diabetic nephropathy is worthy of affirmation. However, this meta-analysis could not provide reliable evidence for its role in regulating inflammatory factors, inhibiting humoral immunity and reducing podocyte apoptosis.

It is worth noting that the results of our meta-analysis showed the significant heterogeneity in 24 h-UTP, ALB, Scr and BUN. In order to explore the source of heterogeneity, we performed subgroup analysis on observation time and drug type. The results showed that the above factors were not the source of heterogeneity, which may be related to the uneven quality and bias of the included literatures and we used random effects model to analyze and interpret all indicators with significant heterogeneity.

The complex chemical ingredients of tripterygium glycosides is easy to produce side effects, and more attention needs to be paid to its potential adverse reactions. The common adverse reactions of tripterygium glycosides were liver damage, menstrual disorder, gastrointestinal reaction and leukopenia. Liver damage is mainly characterized by mild elevation of aminotransferases, which can generally return to normal after liver protection drug treatment. Menstrual disorders often recover after stopping tripterygium glycosides treatment or after using traditional Chinese medicine preparations. Gastrointestinal reactions and leukopenia generally disappear after symptomatic treatment. Our study also showed that the incidence of adverse reactions in the combined treatment group was higher than that in the control group, and the difference was statistically significant (P < 0.05). Therefore, although tripterygium glycosides provides a choice for the clinical treatment of diabetic nephropathy, its side effects cannot be ignored. Clinicians need to make a comprehensive assessment according to the actual situation, if they choose to apply tripterygium glycosides to treat diabetic nephropathy, it is necessary to pay more attention to the possible adverse reactions to patients and give timely intervention.

There are some limitations to this meta-analysis: (1) The included RCTs were mainly in China, and some of the literature only mentioned random assignment without specific description of the method of randomization, and most of the literature did not describe the blinding method, so the quality of the literature was low, which may lead to selection bias or publication bias. (2) The staging of diabetic nephropathy and the different doses of tripterygium glycosides application in the included original literatures may lead to heterogeneity. (3) Some of the original studies did not adequately describe complications, particularly abnormalities in liver function, white blood cells and menstrual disorders. (4) Certain novel drugs, such as dagliflozin and nonnilidone, have not been widespreadly used in the present, and their effects were not included in this study. (5) Lack of data on long-term efficacy: among all the original literatures included, only one study was observed for 12 months, and the other literatures were observed for less than 6 months, the efficacy of long-term use of tripterygium glycosides need to be further confirmed. (6) All of the included subjects were Chinese, and it may be difficult to apply these findings to other populations which will affect the generality of the results.

## Data Availability

The original contributions presented in the study are included in the article/Supplementary Material, further inquiries can be directed to the corresponding author.
