# Supplementary material for: Efficacy and safety of tripterygium glycosides combined with ACEI/ARB on diabetic nephropathy: a meta-analysis
Source: Front Pharmacol. 2025 Jan 17;15:1493590. doi: 10.3389/fphar.2024.1493590 (PMC11782225; doi:10.3389/fphar.2024.1493590)
Supplement: Supplementary file 1 [file DataSheet1.zip › Supplementary material/Search strings.docx]

**For Pubmed, the string used was:**

("Diabetic Nephropathies"[Mesh]) OR (Diabetic Nephropathies[Title/Abstract])) OR (Nephropathies, Diabetic[Title/Abstract])) OR (Nephropathy, Diabetic[Title/Abstract])) OR (Diabetic Nephropathy[Title/Abstract])) OR (Diabetic Kidney Disease[Title/Abstract])) OR (Diabetic Kidney Diseases[Title/Abstract])) OR (Kidney Disease, Diabetic[Title/Abstract])) OR (Kidney Diseases, Diabetic[Title/Abstract])) OR (Diabetic Glomerulosclerosis[Title/Abstract])) OR (Glomerulosclerosis, Diabetic[Title/Abstract])) OR (Intracapillary Glomerulosclerosis[Title/Abstract])) OR (Nodular Glomerulosclerosis[Title/Abstract])) OR (Glomerulosclerosis, Nodular[Title/Abstract])) OR (Kimmelstiel-Wilson Syndrome[Title/Abstract])) OR (Kimmelstiel Wilson Syndrome[Title/Abstract])) OR (Syndrome, Kimmelstiel-Wilson[Title/Abstract])) OR (Kimmelstiel-Wilson Disease[Title/Abstract])) OR (Kimmelstiel Wilson Disease[Title/Abstract]) AND ("Tripterygium"[Mesh]) OR (Tripterygium[Title/Abstract])) OR (Tripterygiums[Title/Abstract])) OR (Tripterygium wilfordii[Title/Abstract])) OR (Tripterygium wilfordius[Title/Abstract])) OR (wilfordius, Tripterygium[Title/Abstract])) OR (Leigong Teng[Title/Abstract])) OR (Leigong Tengs[Title/Abstract])) OR (Teng, Leigong[Title/Abstract])) OR (Tengs, Leigong[Title/Abstract])) OR (Thundergod Vine[Title/Abstract])) OR (Thundergod Vines[Title/Abstract])) OR (Vine, Thundergod[Title/Abstract])) OR (Vines, Thundergod[Title/Abstract])) OR (Tripterygium hypoglaucum[Title/Abstract])) OR (Tripterygium hypoglaucums[Title/Abstract])) OR (hypoglaucums, Tripterygium[Title/Abstract])

**For Web of science,the string used was:**

(Diabetic Nephropathies* OR Nephropathies, Diabetic* OR Nephropathy, Diabetic* OR Diabetic Nephropathy* OR Diabetic Kidney Disease* OR Diabetic Kidney Diseases* OR Kidney Disease, Diabetic* OR Kidney Diseases, Diabetic* OR Diabetic Glomerulosclerosis* OR Glomerulosclerosis, Diabetic* OR Intracapillary Glomerulosclerosis* OR Nodular Glomerulosclerosis* OR Glomerulosclerosis, Nodular* OR Kimmelstiel-Wilson Syndrome* OR Kimmelstiel Wilson Syndrome* OR Syndrome, Kimmelstiel-Wilson* OR Kimmelstiel-Wilson Disease* OR Kimmelstiel Wilson Disease) AND (Tripterygium* OR Tripterygiums* OR Tripterygium wilfordii* OR Tripterygium wilfordius* OR wilfordius, Tripterygium* OR Leigong Teng* OR Leigong Tengs* OR Teng, Leigong* OR Tengs, Leigong* OR Thundergod Vine* OR Thundergod Vines* OR Vine, Thundergod* OR Vines, Thundergod* OR Tripterygium hypoglaucum* OR Tripterygium hypoglaucums* OR hypoglaucums, Tripterygium)

**For CNKI,the string used was:**

( 主题%='糖尿病肾病' or 题名%='糖尿病肾病' ) OR ( 主题%='糖尿病肾脏疾病' or 题名%='糖尿病肾脏疾病' ) OR ( 主题%='糖尿病肾小球硬化症' or 题名%='糖尿病肾小球硬化症' ) AND ( 主题%='雷公藤' or 题名%='雷公藤' )
