# Supplementary figures and images for: Efficacy and safety of tripterygium glycosides combined with ACEI/ARB on diabetic nephropathy: a meta-analysis
Source: Front Pharmacol. 2025 Jan 17;15:1493590. doi: 10.3389/fphar.2024.1493590 (PMC11782225; doi:10.3389/fphar.2024.1493590)

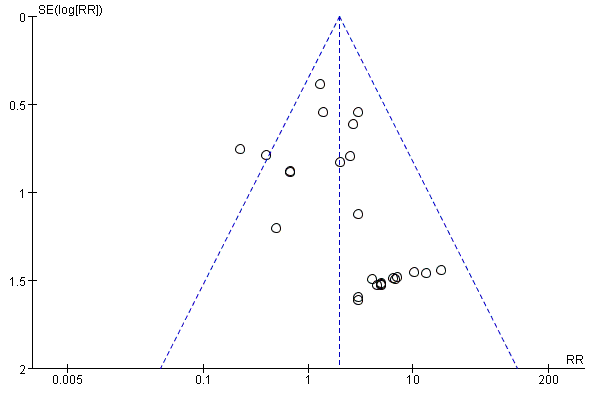

Supplement: Supplementary file 1 [file DataSheet1.zip › Supplementary material/publication bias on adverse reactions.png]

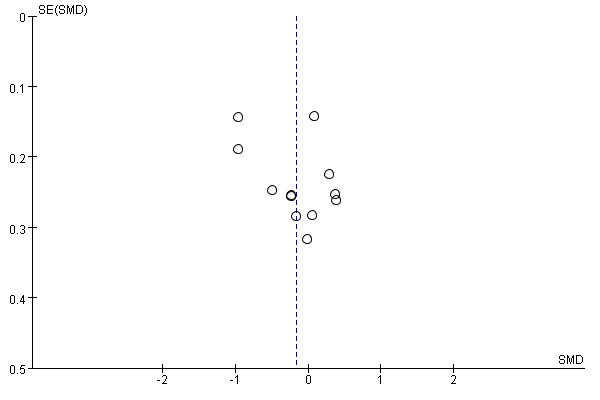

Supplement: Supplementary file 1 [file DataSheet1.zip › Supplementary material/publication bias on blood urea nitrogen.png]

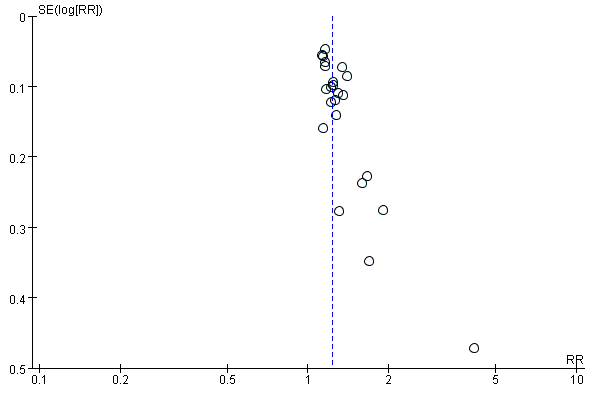

Supplement: Supplementary file 1 [file DataSheet1.zip › Supplementary material/publication bias on effective rate.png]

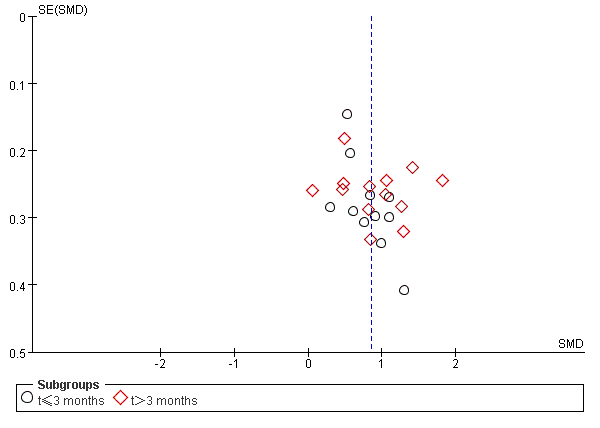

Supplement: Supplementary file 1 [file DataSheet1.zip › Supplementary material/publication bias on serum albumin.png]

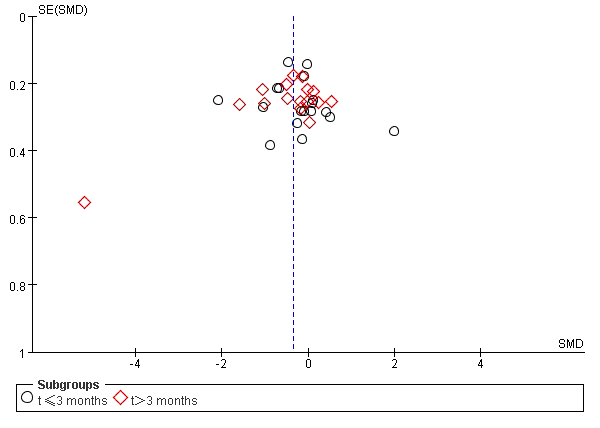

Supplement: Supplementary file 1 [file DataSheet1.zip › Supplementary material/publication bias on serum creatinine.png]
